# Recent advances in the improvement of soybean seed traits by genome editing

**DOI:** 10.5511/plantbiotechnology.23.0610a

**Published:** 2023-09-25

**Authors:** Jaechol Sim, Chikako Kuwabara, Shota Sugano, Kohei Adachi, Tetsuya Yamada

**Affiliations:** 1 Graduate School of Agriculture, Hokkaido University, Kita 9, Nishi 9, Kita-ku, Sapporo, Hokkaido 060-8589, Japan

**Keywords:** CRISPR/Cas9, *Glycine max*, TALEN, transformation, ZFN

## Abstract

Genetic improvement of soybean seed traits is important for developing new varieties that meet the demand for soybean as a food, forage crop, and industrial products. A large number of soybean genome sequences are currently publicly available. This genome sequence information provides a significant opportunity to design genomic approaches to improve soybean traits. Genome editing represents a major advancement in biotechnology. The production of soybean mutants through genome editing is commonly achieved with either an *Agrobacterium*-mediated or biolistic transformation platform, which have been optimized for various soybean genotypes. Currently, the clustered regularly interspaced short palindromic repeat (CRISPR)/CRISPR-associated endonuclease 9 (Cas9) system, which represents a major advance in genome editing, is used to improve soybean traits, such as fatty acid composition, protein content and composition, flavor, digestibility, size, and seed-coat color. In this review, we summarize the recent advances in the improvement of soybean seed traits through genome editing. We also discuss the characteristics of genome editing using the CRISPR/Cas9 system with transformation platforms.

## Introduction

Soybean (*Glycine max*, 2n=2x=40) is a very important crop that is grown worldwide. Its seeds contain high-quality proteins that have an amino acid score comparable to that of beef and egg whites, as well as lipids that are used to prepare vegetable and industrial oil (Liu 2004). Soybean production has increased significantly in response to the increased demand for food, forage crops, and industrial materials (Hartman et al. 2011). The genetic improvement of soybean seed traits is necessary for the development of new varieties that can meet the demands of the industry. Soybean has a paleopolyploid genome, with nearly 75% of the predicted genes present in multiple copies because of the duplicated genome (Schmutz et al. 2010). These redundant sequences pose a challenge to improving the various traits of soybeans. Recently, the advent of genome editing has revolutionized the field of plant genetic engineering by enabling site-directed mutagenesis at targeted genomic sequences. This system is effective for mutagenesis in homologous genes of polyploid plants (Wang et al. 2014). Soybean genome editing may be achieved using three techniques: zinc finger nuclease (ZFN), transcription activator-like effector nucleases (TALENs), and clustered regularly interspaced short palindromic repeat (CRISPR)/CRISPR-associated endonuclease 9 (Cas9) (Cermak et al. 2011; Durai et al. 2005; Jiang et al. 2013). This review summarizes the current research on improving soybean seed traits through genome editing and discusses the various transformation platforms that may be used to effectively implement genome editing in soybean.

## Two transformation platforms in soybean

**Figure 1:**
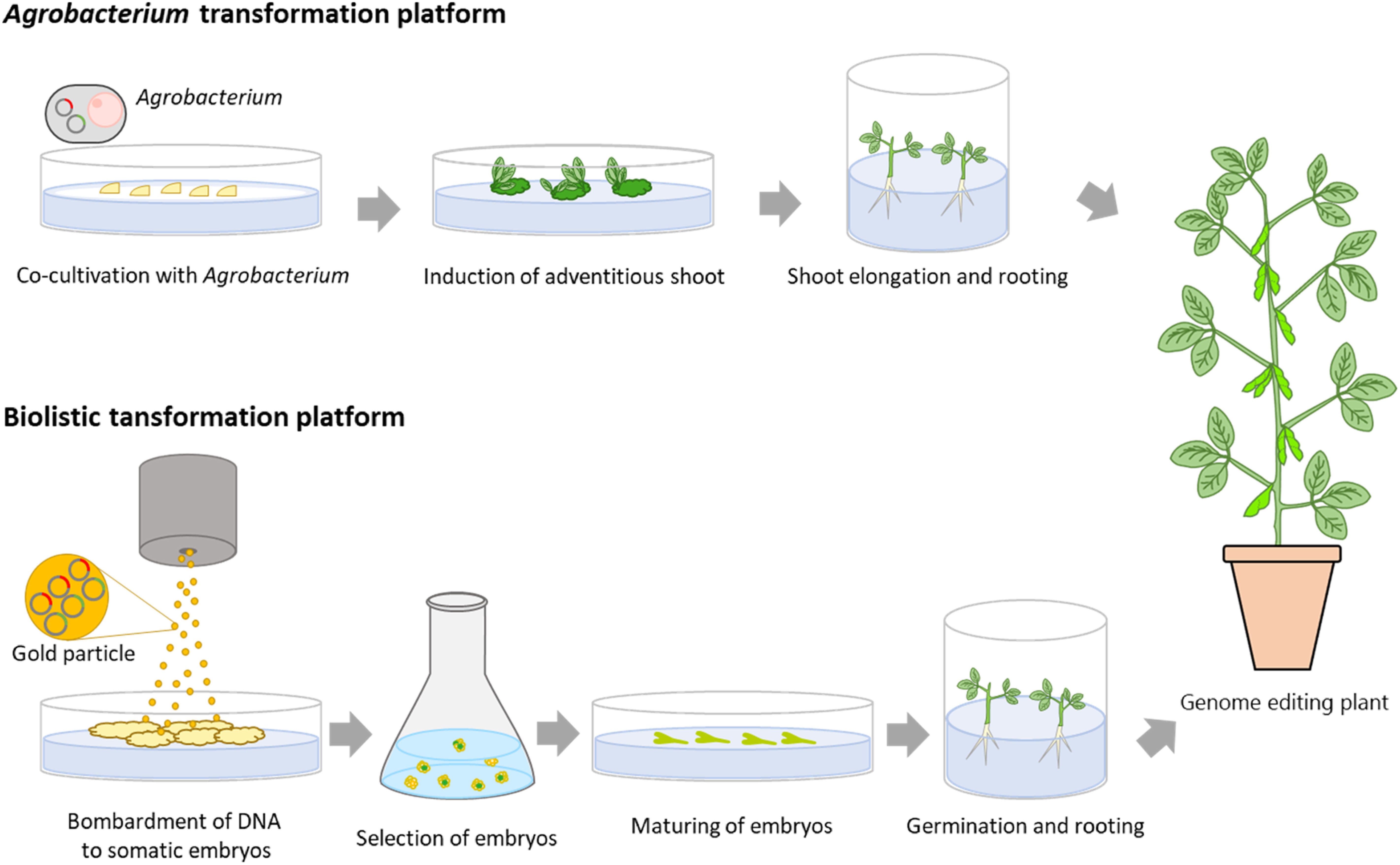
Figure 1. Two major transformation platforms in soybeans. The upper and lower panels show the *Agrobacterium* and biolistic transformation procedures, respectively.

To implement genome editing systems efficiently, the application module of genome editing must be expressed stably in soybean cells. Two reliable transformation methods have been established in soybean, which are shown in Figure 1. The first is a system mediated by *Agrobacterium tumefaciens* as a biological vector. Transgenic soybean plants may be obtained through organogenesis from cotyledonary nodes or immature cotyledons infected with *Agrobacterium* (Hinchee et al. 1988; Parrott et al. 1989a). The cotyledonary node region contains axillary meristems at the junction between the cotyledon and hypocotyl. Successful transformation depends upon the induction of adventitious buds in the cotyledonary node region. In addition, cotyledonary nodes are often prewounded mechanically with a scalpel, small needle, or a stainless-steel microbrush to prepare enough target tissue for *Agrobacterium* infection (Olhoft et al. 2003; Xue et al. 2006; Yamada et al. 2010). Recently, these transformation systems have been applied to various soybean genotypes (Yamada et al. 2012). The *Agrobacterium*-mediated transformation system has also been used for genome editing to improve seed traits using the TALENs and CRISPR/Cas9 systems (Figure 1, Table 1).

**Table 1:** Table 1. Summary of genome editing studies for the improvement of soybean seed traits.

Seed traits	Target gene(s)	Genome editing system	Delivery system	Improved traits and expected benefits by genome editing	Reference
Fatty acid composition	Fatty acid desaturase 2-1A (*FAD2-1A*), *FAD2-1B*	TALEN	Agrobacterium	High oleic acid content	Haun et al. (2014)
	*FAD2-1a*, *FAD2-1b*, *FAD3*	TALEN	Agrobacterium	High oleic acid content	Demorest et al. (2016)
	*FAD2-1a*	ZFN	Biolistic	Not analyzed	Bonawitz et al. (2019)
	*FAD2-2*	CRISPR/Cas9	Agrobacterium	High oleic acid content	Al Amin et al. (2019)
	*FAD2-1a*, *FAD2-1b*	CRISPR/Cas9	Agrobacterium	High oleic acid content	Do et al. (2019)
	*FAD2-1a*, *FAD2-2a*	CRISPR/Cas9	Agrobacterium	High oleic acid content	Wu et al. (2020)
	*FAD2-1b*, *FAD2-2c*	CRISPR/Cas9	Agrobacterium	High oleic acid content	Xiao et al. (2022)
	*FAD2-1a*, *FAD2-1b*	CRISPR/Cas9	Agrobacterium	High oleic acid content	Fu et al. (2022)
Carbon distribution	β-ketoacyl-[acylcarrier protein] synthase 1 (*KASI*)	CRISPR/Cas9	Agrobacterium	High sucrose content	Virdi et al. (2020)
Protein content	ABI3-interacting protein 2 (*AIP2a*, *AIP2b*)	CRISPR/Cas9	Biolistic	High protein content	Shen et al. (2022)
Protein composition	Gly m Bd 28K, Gly m Bd 30K	CRISPR/Cas9	Agrobacterium	Hypoallergenic	Sugano et al. (2020)
	Gly m Bd 30K	CRISPR/Cas9	Biolistic	Hypoallergenic	Adachi et al. (2021)
Flavor	Lipoxygenase (*LOX1*, *LOX2*, *LOX3*)	CRISPR/Cas9	Agrobacterium	Inhibition of lipid peroxidation	Wang et al. (2020)
	Betaine aldehyde dehydrogenase (*BADH2*)	CRISPR/Cas9	Agrobacterium	Granting aroma	Qian et al. (2022)
Digestibility	Galactinol synthase (*GOLS1A*, *GOLS1B*)	CRISPR/Cas9	Agrobacterium	Low oligosaccharides content	Le et al. (2020)
	Raffinose synthase (*RS2*, *RS3*)	CRISPR/Cas9	Agrobacterium	Low oligosaccharides content	Cao et al. (2022)
Phytic acid content	Inositol-pentakisphosphate 1-kinase (*IPK1*)	CRISPR/Cas9	Agrobacterium	Low phytic acid content	Song et al. (2022)
Seed size	PEAPOD (*PPD1*, *PPD2*)	CRISPR/Cas9	Agrobacterium	Big size seed	Kanazashi et al. (2018)
	KINASE-INDUCIBLE DOMAIN INTERACTING 8 (*KIX8*)	CRISPR/Cas9	Agrobacterium	Big size seed	Nguyen et al. (2021)
Seed-coat color	DICER-LIKE2 (*DCL2a*, *DCL2b*)	CRISPR/Cas9	Agrobacterium	Brown seed-coat color	Jia et al. (2020)

The second method is biolistic transformation, in which small tungsten or gold particles coated with the desired genes are delivered to plant cells (Christou et al. 1988). Somatic embryos are often used as an explant for soybean biolistic transformation. Proliferative somatic embryos can retain regenerative potential for more than a year and may be readily induced if necessary (Finer and Nagasawa 1988; Parrott et al. 1988). Because the successful biolistic transformation is closely dependent upon embryogenesis and proliferation of embryonic tissue, the soybean genotypes available for biolistic transformation are more limited compared with those for *Agrobacterium*-mediated transformation (Parrott et al. 1989b; Yamada et al. 2012). However, a biolistic transformation platform has been used for genome editing to improve seed traits using the ZFN and the CRISPR/Cas9 systems (Figure 1, Table 1).

## Improvement of seed traits using the ZFN system

ZFN is a genome editing system that uses a pair of artificial enzymes with a zinc finger domain and DNA cleavage activity (Urnov et al. 2010). The first study using the ZFN system in soybean was the mutagenesis of DICER-LIKE genes (*DCL4a* and *DCL4b*) in a hairy root transformation platform using *A. rhizogenes* dedicated to the rapid confirmation of gene function (Sander et al. 2011). For genome editing of whole soybean plants, fatty acid composition was improved in mature seeds using the ZFN system. Soybean seeds normally contain high levels of polyunsaturated fatty acids, such as linoleic and linolenic acids (Liu 2004). These fatty acids are unstable and susceptible to producing undesirable flavors and trans fatty acids (Liu 2004). Therefore, reducing the content of polyunsaturated fatty acids and increasing monounsaturated fatty acid (oleic acid) content remains an important issue in soybean breeding. Subsequently, mutagenesis of whole soybean plants using the ZFN system was performed on the fatty acid desaturase gene (*FAD2-1a*) encoding ω-6 desaturase, which is involved in the synthesis pathway from oleic acid to linoleic acid (Table 1, Bonawitz et al. 2019). Although the composition of fatty acids in seeds was not determined in this report, other studies using RNA interference (RNAi) to achieve *FAD2-1a* knockdown successfully altered the fatty acid composition of transgenic soybean seeds from 20% oleic acid to 80% (Wagner et al. 2011). Therefore, a similar change in fatty acid composition is expected upon mutagenesis of the *FAD2-1a* gene using the ZFN system. Furthermore, Bonawitz et al. (2019) demonstrated that a 7.1 kb DNA fragment containing the selectable and reporter genes was inserted at the cleavage site of the *FAD2-1a* gene without homology-directed repair (HDR) and this insertion mutation was inherited by the descendants. These results demonstrate that targeted insertion of exogenous nucleotides may be mediated by nonhomologous end joining repair. Despite the successful results described above, there are currently no practical applications for genome-edited soybean using the ZFN system.

## Improvement of seed traits using the TALENs system

TALENs is a genome editing system using a pair of enzymes including the DNA-binding domain of transcription activator-like effector and a DNA cleavage enzyme (Cermak et al. 2011). Two studies were done to improve the fatty acid composition of soybean seeds through mutagenesis using the TALENs system (Table 1). The first soybean study involved the mutagenesis of two *FAD2* genes (*FAD2-1A* and *FAD2-1B*) using the *Agrobacterium*-mediated transformation platform (Haun et al. 2014). Homozygous double-mutants of the *FAD2-1A* and *FAD2-1B* genes exhibited elevated levels of oleic acids from 20% to 80% relative to total fatty acids (Haun et al. 2014). Transgene-free plants that harbored mutant alleles for the targeted *FAD2* loci were also found in the descendants. Demorest et al. (2016) performed mutagenesis using the TALENs system for the *FAD2-1a*, *FAD2-1b*, and *FAD3a* genes using the *Agrobacterium*-mediated transformation platform. The fatty acid profile in mature seeds indicated that the mutant seeds contained a higher oleic acid content compared with the wild-type (Demorest et al. 2016). Triple mutants of the *FAD2-1a*, *FAD2-1b*, and *FAD3a* genes also exhibited higher oleic acid composition (82.2%) compared with that (77.5%) of *FAD2-1a* and *FAD2-1b* double-mutants (Demorest et al. 2016). These mutants showed a significant decrease in polysaturated fatty acids as well as linoleic and linolenic acids in the seeds. Transgene-free segregants were also obtained in the subsequent generation (Demorest et al. 2016). High-oleic soybean developed through site-directed mutagenesis using the TALENs system is the first genome-edited ingredient to be released for consumers in the soybean market.

## Improvement of seed traits by CRISPR/Cas9 system

The CRISPR/Cas9 is a genome editing system consisting of a complex of Cas9 nuclease with a single-guide RNA (sgRNA) (Cong et al. 2013). The first study of the CRISPR/Cas9 system in soybean was performed in a hairy root transformation using *A. rhizogenes* and a somatic embryo using a biolistic transformation platform (Jacobs et al. 2015). Genome editing of whole soybean plants was subsequently performed using a biolistic transformation platform and the induced mutations were inherited by the next generation (Li et al. 2015). Li et al. (2015) also demonstrated that the CRISPR/Cas9 system can be used to insert arbitrary DNA fragments into a targeted region mediated by the HDR mechanism. In this study, transgene-free segregants were obtained in subsequent generations.

The number of studies involving the modification of soybean seed traits using the CRISPR/Cas9 system is considerably larger compared with those using the ZFN or TALENs systems (Table 1). The CRISPR/Cas9 system can be used more frequently for genome editing than the ZFN and TALENs systems due to the ease of vector construction. Among these examples, the largest number of studies have been focused on increasing the oleic acid content in seeds (Table 1). There is a gene family for the *FAD2* gene in the soybean genome and mutagenesis of each *FAD* homolog has been examined in an attempt to increase oleic acid content. The CRISPR/Cas9 modules of the *FAD2-1a* and *FAD2-1b* genes were tested for their mutagenic ability in a transient hairy root transformation using *A. rhizogenes*. Subsequently, the simultaneous mutagenesis of both genes was performed on the *Agrobacterium*-mediated transformation platform (Do et al. 2019). Double homozygous mutant plants exhibited a high oleic acid phenotype (83.3%) compared with the wild-type (20.2%) in mature seeds (Do et al. 2019). Transgene-free double-mutants were also obtained in the subsequent generation (Do et al. 2019). Fu et al. (2022) also demonstrated that mutations in the *FAD2-1a* and *FAD2-1b* genes simultaneously increased oleic acid composition by 85% in double-mutant seeds. Al Amin et al. (2019) showed that mutagenesis of the *FAD2-2* gene results in increased oleic acid content compared with wild-type seeds. Site-directed mutagenesis of the *FAD2-1a* and *FAD2-2a* genes was performed on an *Agrobacterium*-mediated transformation platform (Wu et al. 2020). Double-mutants exhibited increased oleic acid content from 17.10% to 73.50% in the T_2_ generation. Wu et al. (2020) also demonstrated that mutagenesis of the *FAD2-2a* gene contributes to an increase in oleic acid content compared with that of the *FAD2-1a* gene. Mutagenesis of the *FAD2-1b* and *FAD2-2c* genes was also performed on an *Agrobacterium*-mediated transformation platform (Xiao et al. 2022). Double-mutants of the *FAD2-1b* and *FAD2-2c* genes resulted in increased oleic acid content from 19.82% to 54.86% in matured seeds of the T_2_ generation (Xiao et al. 2022). They also demonstrated that there was no significant difference in agronomic traits between the double-mutants and wild-type plants.

Understanding the mechanism of carbon source distribution is very important for soybean breeding, because it is closely associated with the lipid or sugar content in seeds. β-ketoacyl-[acyl carrier protein] synthase 1 (KASI) plays an important role as a fatty acid biosynthetic enzyme and is involved in the conversion of sucrose to fatty acids during soybean seed development (Dobbels et al. 2017). Site-directed mutagenesis of the *KASI* gene by the CRISPR/Cas9 system was performed using the *Agrobacterium*-mediated transformation platform (Virdi et al. 2020). Mature seeds that had homozygous in-frame mutant alleles at the targeted locus exhibited a wrinkled and cracked seed-coat phenotype, but showed an increase in sucrose content from 5.97% to 11.50% as well as a decrease in oil content from 18.76% to 5.63% compared with wild-type seeds (Virdi et al. 2020).

Because soybean is a major protein source for food and livestock feed, genetic enhancement of the protein content is a major goal of soybean breeding. Because of the typical negative correlation between protein and oil content, it is difficult to increase protein content while maintaining seed oil at current levels. ABI3-interacting protein 2 (AIP2), which is an E3-RING ubiquitin ligase, catalyzes the degradation of the abscisic acid insensitive 3 (ABI3) transcription factor (Lara et al. 2003). The regulation of protein accumulation in seed tissue is dependent on ABA levels (Finkelstein et al. 2002). Site-directed mutagenesis of the *AIP2a* and *AIP2b* genes by the CRISPR/Cas9 system was performed using a biolistic transformation platform (Shen et al. 2022). Double-mutant seeds increased protein content by more than 2.0% without significantly altering seed oil content compared with the wild-type (Shen et al. 2022).

Diverse soybean proteins are responsible for the physical properties of soybean products; however, many allergens have also been identified in soybeans. Thus, the production of hypoallergenic soybeans may lead to the expansion of the soybean market. Simultaneous site-directed mutagenesis of two genes encoding the major allergens, Gly m Bd 28K and Gly m Bd 30K, was performed using the *Agrobacterium*-mediated transformation platform (Sugano et al. 2020). Western blot analysis of the allergenic proteins using polyclonal antibodies revealed the disappearance of the targeted allergens in the mature seed of the *Gly m Bd 28K* and *Gly m Bd 30K* double-mutants (Sugano et al. 2020). In addition, whole-genome sequencing confirmed that the *Cas9*-free mutant also lacked an exogenous DNA fragment from the binary vector (Sugano et al. 2020). Adachi et al. (2021) performed site-directed mutagenesis of the *Gly m Bd 30K* gene using a biolistic transformation platform with somatic embryos. In this study, mutation analysis in transgenic embryos revealed that mutations occurred in the target gene immediately after the delivery of the exogenous gene into the embryo cells (Adachi et al. 2021). Transgene-free mutants were obtained in the subsequent generation (Adachi et al. 2021).

Flavor is an important element in processed foods. Hexanal is one of the volatile components produced by a peroxidation reaction of highly unsaturated fatty acids, such as linoleic acid and linolenic acid, and is associated with an undesirable odor characteristic of soybean seeds (Arai et al. 1970). Lipoxygenases (LOXs) are closely associated with the peroxidation of highly unsaturated fatty acids and a deficiency in lipoxygenase activity in soybean seeds decreases the production of hexanal (Matoba et al. 1985). Site-directed mutagenesis of the *LOX1*, *LOX2*, and *LOX3* genes, which are specifically expressed in seed tissue, was performed using the *Agrobacterium*-mediated transformation platform (Wang et al. 2020). A colorimetric assay revealed that triple mutants of the *LOX1*, *LOX2*, and *LOX3* genes were deficient in LOX activity in mature seeds (Wang et al. 2020). Transgene-free mutants were obtained in a later generation. Although the production of hexanal was not examined in this study, these mutants are expected to exhibit decreased hexanal production in seeds. Aromatic vegetable soybean contains a high content of 2-acetyl-1-pyrroline (2AP) as a major volatile compound. A single nucleotide polymorphism in the betaine aldehyde dehydrogenase gene (*BADH2*), which was identified in natural soybean variations in soybean, and is closely involved in the accumulation of 2AP in soybean seeds (Juwattanasomran et al. 2011). Site-directed mutagenesis of the *BADH2* gene was performed using the *Agrobacterium*-mediated transformation platform (Qian et al. 2022). Mutant plants exhibited an increase in 2AP content from 0.81 µg g^−1^ to 7.72 µg g^−1^ in seeds compared with the wild-type seeds (Qian et al. 2022).

Sucrose, raffinose, and stachyose are the major water-soluble carbohydrates present in soybean seeds (Kennedy et al. 1985). Raffinose and stachyose are classified as raffinose family oligosaccharides (RFOs). Because RFOs are indigestible by humans and other monogastric animals, it is desirable to remove RFOs from seeds in soybean breeding programs. Galactinol synthase (GOLS) plays an important role in carbon source distribution between RFOs and sucrose (Taji et al. 2002). Site-directed mutagenesis of the *GOLS1A* and *GOLS1B* genes was performed using the *Agrobacterium*-mediated transformation platform (Le et al. 2020). In double-mutants for both genes, stachyose content was decreased by 35.4%, whereas raffinose content was increased by 41.7% compared with mature wild-type seeds (Le et al. 2020). The quantitative analysis of seed carbohydrates in double-mutants showed a decrease in total RFO content from 64.7 mg g^−1^ (wild-type) to 41.95 mg g^−1^ of dry weight (Le et al. 2020). Transgene-free mutants were also obtained in the subsequent generation (Le et al. 2020). Simultaneous site-directed mutagenesis of the raffinose synthase (*RS*) genes, *RS2* and *RS3*, also resulted in a low content of RFOs in mature seeds of the mutant soybean plants (Cao et al. 2022). The *RS2* and *RS3* double-mutants exhibited increased sucrose content and decreased raffinose and stachyose content in mature seeds (Cao et al. 2022). In this study, the polycistronic expression of multiple gRNAs was performed using the tRNA processing system of the host plant cells (Cao et al. 2022). Transgene-free mutants were also obtained in the subsequent generation.

Phytic acid (PA) represents a major form of phosphorus in soybean seeds. PA is not available to monogastric animals that lack phytase in their digestive tract (Erdman 1979). A reduction of PA content in soybean seeds is necessary, because undigested PA phosphorous in animal waste is a major cause of environmental pollution (Shi et al. 2007). Inositol-pentakisphosphate 1-kinase (IPK1) catalyzes the last step of PA biosynthesis. Site-directed mutagenesis of the *IPK1* gene in soybean was performed using the *Agrobacterium*-mediated transformation platform (Song et al. 2022). Site-directed mutagenesis of the *IPK1* gene resulted in a 20% reduction in PA content in soybean seeds compared with the wild-type (Song et al. 2022).

Seed size is one of the most important characteristics in soybean food processing. Mutations in the PEAPOD (*PPD*) ortholog have resulted in gigantism of multiple organs, including legume seeds (Ge et al. 2016; Naito et al. 2017). Site-directed mutagenesis of the *PPD1* and/or *PPD2* genes was performed using the *Agrobacterium*-mediated transformation platform (Kanazashi et al. 2018). The soybean mutant that carried a frameshift mutation at one locus and an in-frame mutation at the other locus exhibited increased seed size compared with the wild-type (Kanazashi et al. 2018). A Kinase-Inducible Domain Interacting 8/9 (KIX8/9) is also closely associated with the control of seed size. The KIX8/9 protein is known to interact with PPD1/2 and MYC3/4 (Liu et al. 2020). This complex regulates the expression of Growth-Regulating Factor (GRF)-Interacting Factor 1 (GIF1), which is a transcriptional co-activator involved in plant cell proliferation (Liu et al. 2020). Site-directed mutagenesis of the *KIX8-1* gene was performed using the *Agrobacterium*-mediated transformation platform (Nguyen et al. 2021). Mutant plants showed an increase in seed size in mature seeds (Nguyen et al. 2021).

Seed color is closely associated with various processing applications of soybeans (Hwang et al. 2020). Yellow soybeans, which are currently the predominant soybean variety in the world, do not contain pigmentation in the seed coat. Inhibition of seed-coat pigmentation results from RNA silencing of chalcone synthase (CHS), which is a key enzyme for the biosynthesis of flavonoids (Senda et al. 2004; Tuteja et al. 2004). This gene silencing is enhanced by 22-nucleotide small RNAs, which trigger the production of secondary small interfering RNAs (siRNA). Dicer-Like 2 (DCL2) plays an important role in the production of 22-nucleotide siRNA. Site-directed mutagenesis of the *DCL2a* and *DCL2b* genes was performed using the *Agrobacterium*-mediated transformation platform (Jia et al. 2020). Double-mutants for the *DCL2a* and *DCL2b* genes increased the expression of *CHS* mRNA, which was downregulated in the seed coat (Jia et al. 2020). An increase in the expression of the *CHS* genes resulted in a change in seed-coat color from non-pigmented to brown (Jia et al. 2020).

There are many soybean plants in which various seed components have been improved by genome editing. However, none are available on the soybean market except for the high-oleic soybean mentioned above. Successfully bringing genome-edited soybean to the soybean market may require site-directed mutagenesis against the most suitable genotype for release as a new soybean variety, in addition to achieving social consensus for genome-edited crops. Moreover, it may be necessary to generate a breakthrough mutant that will trigger the active use of soybean genome-edited plants.

## CRISPR/Cas9 system in two transformation platforms

We performed site-directed mutagenesis of soybeans with a single gRNA for the Gly m Bd 30K locus using the *Agrobacterium*-mediated and biolistic transformation platforms (Adachi et al. 2021; Sugano et al. 2020). These transformation platforms involved different processes and conditions in tissue culture and the selection of transgenic cells and plants (Figure 1, Table 2). These platforms also showed different characteristics in site-directed mutagenesis (Table 2). For site-directed mutagenesis using the *Agrobacterium*-mediated transformation platform, there are often three or more alleles for a single locus in the T_1_ generation (Sugano et al. 2020). However, the problem of mosaicism in mutagenesis was resolved by advancing the generation (Sugano et al. 2020). The chimeric mutations in the T_1_ generation have been identified in other site-directed mutagenesis studies that we have previously conducted using the *Agrobacterium*-mediated transformation platform (Kanazashi et al. 2018). On the other hand, only a biallelic mutation was identified in the targeted locus of transgenic embryo lines obtained using the biolistic transformation platform (Adachi et al. 2021). The same mutant alleles were subsequently detected in the T_0_ plants generated from transgenic embryos and these mutant alleles were inherited by the subsequent generation (Adachi et al. 2021). These results indicate that mutations were introduced in the target gene immediately after the introduction of the exogenous gene into embryo cells. In soybean site-directed mutagenesis, the introduction of the CRISPR/Cas9 module into somatic embryos and a high selection pressure of transformed cells may induce stable and rapid mutagenesis in the target gene of the T_0_ generation.

**Table 2:** Table 2. Site-directed mutagenesis of the *Gly m Bd 30K* gene using the CRISPR/Cas9 system performed with two soybean transformation platforms.

	*Agrobacterium*	Biolistic
Explants	Cotyledonary nodes	Somatic embryos
Soybean genotypes	Enrei, Kariyutaka	Jack
Number of explants per experiment	100∼200	Many
Selectable agents of transgenic plants	Glufosinate	Hygromycin
Period until generating T_0_ plants	2 months~	3 months~
Mosaicism of mutations in T_1_ and T_2_ plants	Frequent	Rare
Nucleotide characteristics in the mutant allele	Mainly deletion or insertion from one to ten-nucleotides	Deletion or insertion from one to ten-nucleotides, and insertion of more than 100-nucleotides
Frequency of obtaining Cas9-free plants in the later generations	High frequent	High frequent

For site-directed mutagenesis using the biolistic transformation platform, unintentional DNA insertions of 600- or 133-nucleotides were detected in the targeted locus of transgenic embryo lines (Adachi et al. 2021). These fragments may be derived from the exogenous gene during site-directed mutagenesis (Adachi et al. 2021). On the other hand, no large fragment insertion was detected in the soybean mutants generated by site-directed mutagenesis using the *Agrobacterium*-mediated transformation (Sugano et al. 2020). These results indicate that fragmentation of foreign genes and unintended DNA insertion into target genes can readily occur in the process of site-directed mutagenesis by biolistic transformation.

The modification of soybean seed traits by genome editing using the CRISPR/Cas9 system has had a significant impact on soybean farmers, users, and consumers. However, this system requires further optimization in several aspects. The development of genotype-independent tissue-culture methods and the innovative introduction methods in genome editing will greatly advance soybean research.
